# The moderation of satisfaction with working conditions in the association between workload and mental health among healthcare workers collecting test samples in the post-COVID-19 era

**DOI:** 10.3389/fpubh.2023.1106299

**Published:** 2023-06-08

**Authors:** Kehui Wang, Bin Yang, Cuiping Wu, Lianxue Zheng

**Affiliations:** ^1^Faculty of Education, Henan University, Kaifeng, Henan, China; ^2^The General Office, The First Affiliated Hospital of Henan University of Traditional Chinese Medicine, Zhengzhou, Henan, China; ^3^Department of Occupational and Environmental Health, College of Public Health, Zhengzhou University, Zhengzhou, Henan, China; ^4^Department of Epidemiology and Biostatistics, College of Public Health, Zhengzhou University, Zhengzhou, Henan, China

**Keywords:** anxiety disorder, depression, somatization, satisfaction with working conditions, workload, healthcare workers, COVID-19

## Abstract

**Background:**

This study aimed to examine the associations between workload and satisfaction with working conditions and mental health (i.e., anxiety disorder, depression, and somatization) of healthcare workers collecting test samples during the local outbreaks of COVID-19, and explore satisfaction with working conditions as a moderator of these relationships.

**Methods:**

A total of 1,349 participants were obtained via an online survey in Zhengzhou, Henan Province, China. Multivariate regression was used to assess the associations between workload and satisfaction with working conditions and anxiety disorder, depression, and somatization. The simple slope analysis and Johnson-Neyman technique were used to assess the effect value and change trend of the moderator.

**Results:**

The prevalence of anxiety disorder, depression, and somatization were 8.6, 6.9, and 19.2% of healthcare workers collecting test samples, respectively. High levels of workload were associated with an increased risk of an anxiety disorder (OR = 1.81, 95%CI = 1.17–2.78), depression (OR = 1.92, 95%CI = 1.19–3.10), and somatization (OR = 1.90, 95%CI = 1.40–2.57), while high satisfaction of working conditions was associated with a reduction in the risk of these outcomes, and ORs (95%CI) were 0.35 (0.20–0.64), 0.27 (0.13–0.56), and 0.32 (0.21–0.48), respectively. The findings also indicated that a weaker association between workload and anxiety disorder, as well as depression and somatization, has been reported in those with a high level of satisfaction with working conditions.

**Conclusion:**

Workload significantly increased the risk of healthcare workers suffering from psychological problems, while satisfaction with working conditions alleviated these negative effects, and effective resource support was crucial for healthcare workers.

## 1. Introduction

The COVID-19 pandemic has presented an unprecedented challenge to healthcare workers (HCWs) (1). Studies indicated that healthcare workers reported poorer health outcomes and greater exposure to psychosocial risks (2). Studies also found that healthcare workers had experienced serious anxiety, depression, insomnia, somatization, post-traumatic stress disorder, and other mental problems during the pandemic (3). The sources of psychological challenges for healthcare workers include not only the explosive increase of workload in the short term, but also fears about infection or contagion for families, insufficient personal protective equipment (PPE), and poor working environment conditions (4, 5), among which work overload was a key source affecting physical and mental health (6). For example, many frontline healthcare workers work more than 16 h a day on average to care for patients infected by COVID-19 during the outbreak of the pandemic (7).

In the post-pandemic era, local outbreaks and distributions of COVID-19 have become a new pandemic pattern. When positive patients appeared in a region or city, nucleic acid testing of close contacts or indirect possible contacts was an important strategy for the early detection of positive patients. Notably, the incubation period of the virus or errors in the test may lead to a misleading result in a single test and an increased risk of epidemic spread. Therefore, repeated nucleic acid sampling and testing have become the primary strategy for the identification of positive cases. In addition, the increased rate of transmission of mutated viruses in the population means that nucleic acid sample collectors need to complete the testing of millions of people in a short period to identify infected individuals in a timely manner, which was undoubtedly a huge challenge for healthcare workers.

The Job Demand—Control Model (JD-C) believed that work stress comes from the joint influence of characteristics that the job demands and job control (8). Job demand refers to the factors existing in the work situation that reflect the number and difficulty of work tasks undertaken by employees, mainly including workload, role conflict, and problem-solving requirements (8), where the workload, referred to the amount of work performed or capable of being usually performed within a specific period, was considered the most important work predictor of psychosocial effect (e.g., working pressure, burnout, and anxiety) (9–11). The Job Demands-Resources Model (JD-R) suggested that work resources refer to the factors that help workers achieve their work objectives and reduce work requirements and physical and mental consumption (12), such as autonomy, social support, working conditions, rewards, and development opportunities (13). The buffering hypothesis of the JD-R theory proposes an interactive effect, that is, various resources buffer the pressure and mental problems caused by various demands, and the results were partly verified in previous studies (14). Working conditions, one of the job resources provided by managers, referred to working facilities, the working system, and the working environment and might be an important predictor of employees' mental health problems. Studies have suggested that positive measures in the workplace can improve the working life of employees, which in turn can improve mental wellbeing even though they are not explicitly mental health support services (1, 15). Satisfaction with working conditions (SWC) shows the subjective feeling of working resources and more directly reflects the psychological status of employees. Studies have shown that job satisfaction was not only a predictor of great mental health (16) but also moderated the relationship between work and mental health (17). Therefore, based on previous research evidence, we propose two hypotheses: Hypothesis 1: workload negatively predictes mental problems, while satisfactory working conditions predicts positive mental states. Hypothesis 2: satisfaction with working conditions moderates the relationship between working conditions and mental health.

The present study aimed to examine workload and satisfaction with working conditions and mental health (i.e., depression, anxiety, and somatization) and explore satisfaction with working conditions as a moderator of these relationships.

## 2. Materials and methods

### 2.1. Participants

The data of the present study were obtained from an online survey conducted in Zhengzhou, Henan Province of China, from 1 September 2021 to 7 September 2021 when a wave of local epidemics had just been confined in Henan Province. The participants of this study were healthcare workers who participated in nucleic acid detection when a local epidemic occurred. The questionnaire was distributed via an online platform, called www.wjx.cn. First, a questionnaire introduction and explanation document were provided to indicate the purpose and object of this survey. Then, participants who met the inclusion criteria and were willing to participate in the survey could complete and submit the questionnaire through the link in the attachment. To ensure the quality of the questionnaire, we filled out and estimated the time to complete the questionnaire in advance, and then the time spent by participants filling out the questionnaire was used to evaluate the quality of the questionnaire. Those participants who completed the questionnaire in <180 s were excluded, and a total of 1,349 valid questionnaires were obtained for analysis (88.2% were women).

This study was following the ethical standards of the responsible committee on human experimentation, and all participants read the instructions and informed consent before filling in the questionnaire. Participants could stop answering and quit at any time if they were unwilling to continue answering.

### 2.2. Measurement

#### 2.2.1. Dependent variables

##### 2.2.1.1. Anxiety disorder

The 20-item Self-rating Anxiety Scale (SAS) was used to assess anxiety disorder over the previous 2 weeks (18) in terms of somatic (e.g., arm and leg shaking and trembling) and psychological (e.g., feeling afraid for no reason) symptoms. Responses were given on a 4-point scale, and scores ranged from 1 (none, or a little of the time) to 4 (most, or all of the time). The total score of the SAS ranged from 20 to 80, which was converted to an index score with a potential range of 25 to 100, and an index score of ≥50 was classified as an anxiety disorder (19). Cronbach's alpha in the present survey was 0.800.

##### 2.2.1.2. Depression

The 9-item Patient Health Questionnaire (PHQ-9) was used to assess depression (20). The scale consists of nine items that measure the frequency of depressive symptoms, such as depressed or desperate, over the last 2 weeks. Responses were given on a 4-point scale, and scores ranged from 0 (not at all) to 3 (nearly every day). The total score of the PHQ-9 ranged from 0 to 27, and scores ranging from 0 to 4, 5 to 9, 10 to 14, and 15 or above are classified as minimal, mild, moderate, and severe depression, respectively. In this study, the total score, ≥10 were classified as depression. Cronbach's alpha in the present survey was 0.902.

##### 2.2.1.3. Somatization

The 15-item Patient Health Questionnaire (PHQ-15) was employed to assess the severity of somatization (21). The scale consists of 15 items that asked whether somatic symptoms, such as stomach pain, constipation or diarrhea, back pain, headache, chest pain, feeling heart pound or race, and dizziness, were present in the last 4 weeks with varying levels of severity. Responses were given on a 4-point scale, and scores ranged from 0 (not bothered at all) to 2 (bothered a lot). The total score of the PHQ-15 ranged from 0 to 30, and scores ranging from 0 to 4, 5 to 9, 10 to 14, and 15 or above are classified as minimal, low, medium, and severe somatic symptoms, respectively. We divided the total score, and scores ≥10 were classified as somatization. Cronbach's alpha in the present study was 0.865.

#### 2.2.2. Independent variables

##### 2.2.2.1. Workload

In this study, the workload of healthcare workers was measured by the number of tasks and continuous working time (22), which were measured using two items, respectively: “How many waves of sampling did you take part in during this local outbreak?” and “How often do you work shifts?”. We define working tasks as mild (once), moderate (twice), and severe (three or more times) according to the answers given by the participants during sampling, and scores range from 1 (mild) to 3 (severe). Available options for shift time were short (3 h or less), moderate (4–6 h), and long (7 h or more), and scores range from 1 (short) to 3 (long). The sum of the scores of the two items was used to evaluate the workload of the healthcare worker. The *Pearson* correlation coefficient of the two items was 0.122 (*p* < 0.05). In addition, we further divided the workload into a binary variable, if any variable reported as “severe” or “long” of the number of participating waves and shift time, then it was divided into high workload (coded as 1), and others were divided into low workload (coded as 0).

##### 2.2.2.2. Satisfaction with working conditions

**Figure 1:**
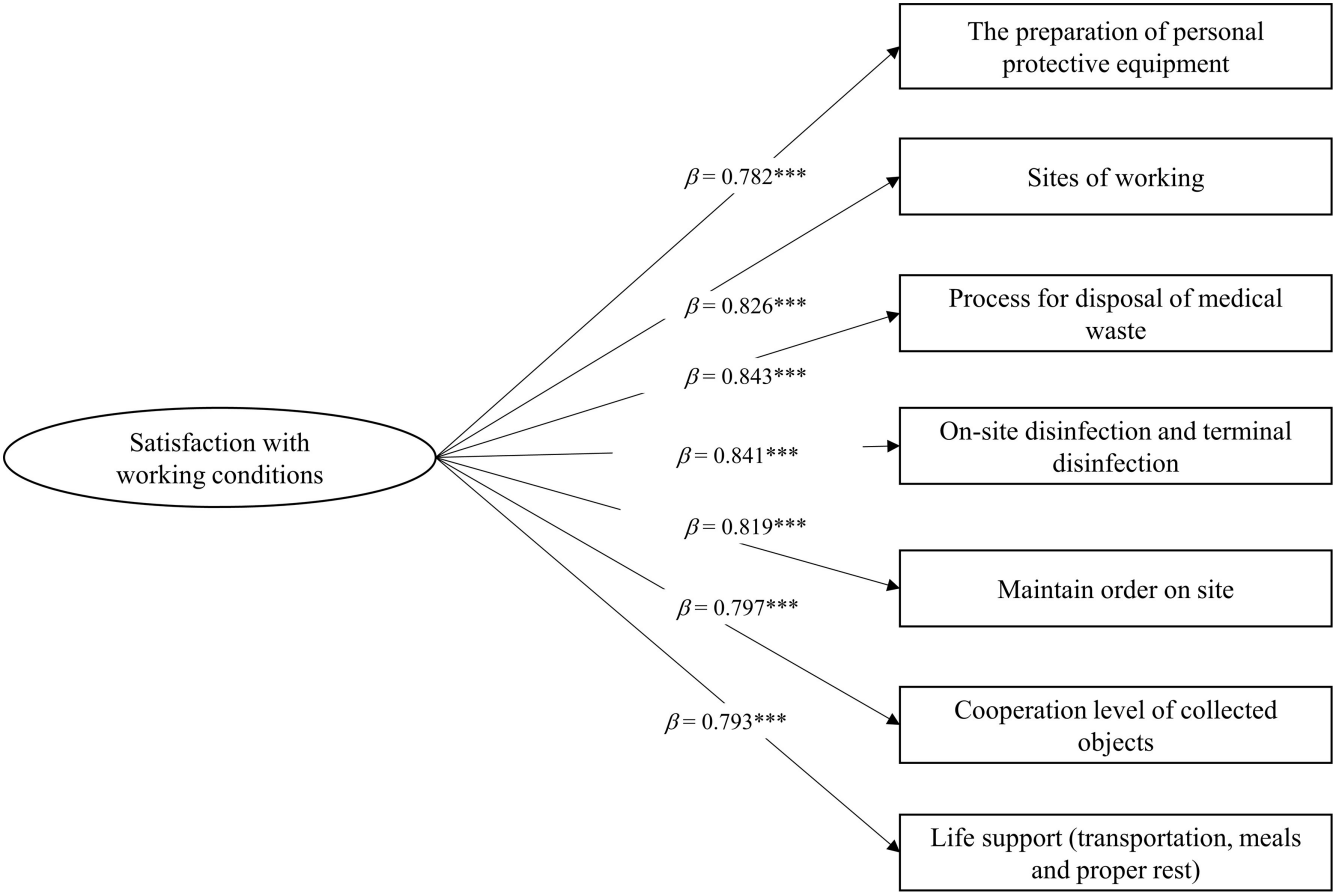
Standardized factor loadings of satisfaction with working conditions latent factor items. ***p < 0.001.

Satisfaction with the working condition was accessed via seven items (preparation of personal protective equipment, sites of work, the process for disposal of medical waste, on-site disinfection and terminal disinfection, maintaining order on-site, life support (transportation, meals, and proper rest), and cooperation level of collected objects). Healthcare workers reported their satisfaction with these conditions during their work. Responses were given on a 5-point scale, and scores ranged from 1 (very dissatisfied) to 5 (very satisfied). A confirmatory factor analysis using SPSS AMOS 25.0 (IBM Corporation, Armonk, NY, USA) revealed that four of the seven items loaded significantly onto one factor (Figure 1). Model fitting index: CMIN/DF = 0.272, *p* > 0.05, CFI = 1.00, TLI = 1.00, RMSEA = 0.001, SRMR = 0.002. Satisfaction with working condition latent factor was used for subsequent analyses. Satisfaction with working conditions was measured by the total score of the seven items. Cronbach's alpha in the present study was 0.938. The last 27th percentile of the total score was further divided into a high group and defined as high satisfaction (coded as 1), and others were divided into low satisfaction (coded as 0).

#### 2.2.3. Covariates

Variables including age, gender (male vs. female), occupation (physician vs. nurse), marriage status, self-rated health, and prior participation in the first-line anti-epidemic were considered in the study. Self-rated health was assessed by an item: “How do you evaluate your health?”. Available options ranged from 1 (poor) to 4 (great). We assessed whether health workers participated in the first wave outbreak in early 2020, and the responses were divided into two categories (yes or no).

### 2.3. Statistical analysis

Sample characteristics, such as gender, occupation, age, marriage status, self-rated health, and prior participation in the first-line anti-epidemic were described using n (%), and continuity variables (workload, satisfaction with working conditions, anxiety disorder, depression, and somatization) were described using mean ± standard deviation (*M* ± *SD*). First, binary logistic regression analysis was used to evaluate the associations between workload and satisfaction and outcomes (anxiety disorder, depression, and somatization). Two models were constructed to explore these effects: Model 1 was the crude model and no variables were adjusted, and Model 2 was an adjusted model of controlling for age, gender, marriage status, self-rated health, and prior participation in the first-line anti-epidemic. Next, multiple linear regression was used to assess the relationships between workload and satisfaction with working conditions with outcomes (anxiety disorder, depression, and somatization) after adjusting for a series of covariates, and moderation of the relationship between workload and mental health outcomes (anxiety disorder, depression, and somatization) by satisfaction with working conditions was assessed using regression models with an interaction term (workload x satisfaction with working conditions). Finally, a simple slope analysis was used to assess the associations between workload and psychological outcomes, respectively for low and high SWCs (mean ± one SD, respectively). Furthermore, the Johnson-Neyman method was used to assess trends in the effects of workload on mental outcomes at different values of moderating variables (23).

The variables of workload and satisfaction with working conditions were transformed into z-scores before the regression analysis. All statistical analyses were performed using SPSS 25 (IBM Corporation, Armonk, NY, USA), and PROCESS v3.3 (24) was used to conduct the simple slope analysis and Johnson-Neyman analysis using a significance level of *p* < 0.05.

## 3. Results

Among the 1,349 participants, 88.2% were women and more than half were between 20 and 40 years old. More than half of the participants (78.0%) were nurses, and 54.0% of the participants had participated in the first-line anti-epidemic (Table 1). The results showed that 8.6%, 6.9%, and 19.2% of healthcare workers collecting test samples during the local outbreaks of COVID-19 reported anxiety disorder, depression, and somatization, respectively. Approximately 27.9% of healthcare workers reported high satisfaction with working conditions.

**Table 1:** Sociodemographic characteristics of frontline nucleic acid sampling personnel during the local outbreak of COVID-19 (*n* = 1,349).

**Characteristics**	***N* (%)**	**95% CI**
**Gender**		
Male	159 (11.8)	10.1–13.5
Female	1,190 (88.2)	86.5–89.9
**Age, years**		
≤25	168 (12.5)	10.7–14.2
26~	499 (37.0)	34.4–39.6
31~	429 (31.8)	29.3–34.3
36~	253 (18.7)	16.7–20.8
**Occupation**		
Physician	297 (22.0)	19.8–24.2
Nurse	1,052 (78.0)	75.8–80.2
**Participating in the first-line anti-epidemic before**		
No	620 (46.0)	43.3–48.6
Yes	729 (54.0)	51.4–56.7
**Education level**		
College	199 (14.8)	12.9–16.6
Bachelor	977 (72.4)	70.0–74.8
Master and above	173 (12.8)	11.0–14.6
**Marriage status**		
Unmarried	508 (37.7)	35.1–40.2
Married	841 (62.3)	59.8–64.9
**Self-rated health**		
Bad	44 (3.3)	2.3–4.2
Moderate	173 (12.8)	11.0–14.6
Good	1,132 (83.9)	82.0–85.9
Workload, *mean* (SD)	4.15 (0.97)	4.10–4.20
High-workload, *n* (%)	739 (54.8)	52.1–57.4
Satisfaction with working conditions, *mean* (SD)	29.27 (5.33)	28.99–29.56
High-satisfaction, *n* (%)	376 (27.9)	25.5–30.3
Anxiety disorder, *mean* (SD)	41.22 (7.38)	40.82–41.61
Anxiety disorder, *n* (%)	116 (8.6)	7.1–10.1
Depression, *mean* (SD)	3.22 (4.07)	3.00–3.44
Depression, *n* (%)	93 (6.9)	5.5–8.2
Somatization, *mean* (SD)	4.79 (5.38)	4.50–5.08
Somatization, *n* (%)	259 (19.2)	17.1–21.3

CI, Confidence Intervals; SD, standard deviation.

The results showed that a high level of workload significantly increased the risk of an anxiety disorder (OR = 1.81, 95% CI = 1.17–2.78), depression (OR = 1.92, 95% CI = 1.19–3.10), and somatization (OR = 1.90, 95% CI = 1.40–2.57), while high level of satisfaction reduced these risks, and OR and 95% CI were 0.35 (0.20–0.64), 0.27 (0.13–0.56), and 0.32 (0.21–0.48) after adjusting for gender, age, occupation, participating in the first-line anti-epidemic, education level, married status, and self-rated health (Table 2).

**Table 2:** Logistic analysis of workload and satisfaction with working conditions on anxiety disorder, depression, and somatization.

	**Characteristics**		***OR*(95% CI)**	
		**Anxiety disorder**	**Depression**	**Somatization**
Model 1	Workload [ref: low workload]	1.96 (1.29–2.96)	2.04 (1.28–3.24)	2.02 (1.51–2.70)
	SWC [ref: low satisfaction]	0.34 (0.19–0.60)	0.27 (0.13–0.54)	0.33 (0.22–0.48)
Model 2	Female [ref: males]	1.30 (0.55–3.05)	1.43 (0.57–3.61)	1.96 (1.09–3.53)
	Age, years [Ref: ≤ 25]			
	26~	2.40 (1.08–5.36)	1.16 (0.55–2.43)	1.30 (0.78–2.14)
	31~	2.58 (1.05–6.34)	0.95 (0.40–2.26)	1.13 (0.63–2.02)
	36~	1.36 (0.49–3.83)	0.60 (0.22–1.66)	0.98 (0.21–1.87)
	Nurse [ref: physician]	2.13 (1.00–4.52)	1.50 (0.70–3.23)	1.17 (0.74–1.87)
	Participating in the first-line anti-epidemic [ref: no]	1.08 (0.72–1.61)	1.00 (0.65–1.56)	1.39 (1.03–1.87)
	Education level [ref: college]			
	Bachelor	0.39 (0.24–0.63)	0.47 (0.28–0.79)	0.45 (0.31–0.66)
	Master and above	0.51 (0.21–1.25)	0.53 (0.20–1.40)	0.53 (0.28–0.99)
	Married status [ref: unmarried]	0.83 (0.51–1.36)	1.05 (0.61–1.82)	1.02 (0.71–1.48)
	Self-rated health [ref: bad]			
	Moderate	0.35 (0.15–0.79)	0.24 (0.10–0.58)	0.50 (0.24–1.00)
	Good	0.20 (0.09–0.41)	0.17 (0.08–0.37)	0.25 (0.13–0.48)
	Workload [ref: low workload]	1.81 (1.17–2.78)	1.92 (1.19–3.10)	1.90 (1.40–2.57)
	SWC [ref: low satisfaction]	0.35 (0.20–0.64)	0.27 (0.13–0.56)	0.32 (0.21–0.48)

Model 1 means crude model; Model 2 adjusted for gender, age, occupation, participating in the first-line anti-epidemic, education level, married status, and self-rated health. CI, Confidence Intervals; SWC, Satisfaction with working conditions.

The results also suggested that women reported a higher risk of somatization than men (OR = 1.96, 95% CI = 1.09–3.53). Healthcare workers aged 26–30 years old (OR = 2.40, 95% CI = 1.08–5.36) and 31–35 years old (OR = 2.58, 95% CI = 1.05–6.34) reported a higher risk of anxiety disorder. The results also suggested that participants who participated in the first-line anti-epidemic before reported a higher risk of somatization (OR = 1.39, 95% CI = 1.03–1.87). Compared with the education level of college, those with the education level of bachelor's degree and master's degree or above reported lower odds of anxiety disorder, depression, and somatization, but for anxiety disorders and depression, the statistical significance only was observed in the education level of bachelor's degree, and OR (95%CI) were 0.39 (0.24–0.63) and 0.47 (0.28–0.79). In addition, participants with moderate and good self-reported health were more likely to report a lower risk of anxiety disorder, depression, and somatization than those who had poor health.

Similar to logistic regression analysis, multiple-line regression results showed that workload positively predicted anxiety disorder (*b* = 0.73, *p* < 0.001), depression (*b* = 0.39, *p* < 0.001), and somatization (*b* = 0.88, *p* < 0.001) in healthcare workers, even after adjusting a series of covariates, while satisfaction with working conditions was negatively associated with anxiety disorder, depression, and somatization (Model 1 in Table 3). Three multiple regression models that included interactive items of satisfaction with working conditions and workload were run to assess satisfaction with working conditions as a potential moderator (Model 2 in Table 3). The results showed that workload × satisfaction with working conditions was negatively correlated with an anxiety disorder (*b* = −0.35, *p* = 0.075), depression (*b* = −0.19, *p* = 0.067), and somatization (*b* = −0.27, *p* = 0.043).

**Table 3:** Multiple linear analysis of workload and satisfaction with working conditions on anxiety disorder, depression, and somatization.

**Model**	**Variables**	**Anxiety disorder**			**Depression**			**Somatization**		
		***b***	***t***	***P***	***b***	***t***	***P***	***b***	***t***	***P***
Model 1	Workload	0.73	3.607	<0.001	0.39	3.706	<0.001	0.88	6.438	<0.001
	SWC	−1.30	−6.441	<0.001	−1.31	−12.410	<0.001	−1.66	−12.138	<0.001
	*F*	14.241			31.717			38.454		
	*R* ^2^	0.087			0.176			0.205		
Model 2	Workload	0.75	3.734	<0.001	0.40	3.836	<0.001	0.90	6.580	<0.001
	SWC	−1.26	−6.219	<0.001	−1.29	−12.146	<0.001	−1.63	−11.866	<0.001
	Workload × SWC	−0.35	−1.782	0.075	−0.19	−1.832	0.067	−0.27	−2.027	0.043
	*F*	13.156^***^			28.931^***^			35.100^***^		
	*R* ^2^	0.090			0.178			0.208		

^***^p < 0.001. All models adjusted for gender, age, occupation, participation in the first-line anti-epidemic, education level, married status, and self-rated health. SWC, Satisfaction with working conditions. The workload and SWC were transformed into z-scores for moderating effect analysis.

**Figure 2:**
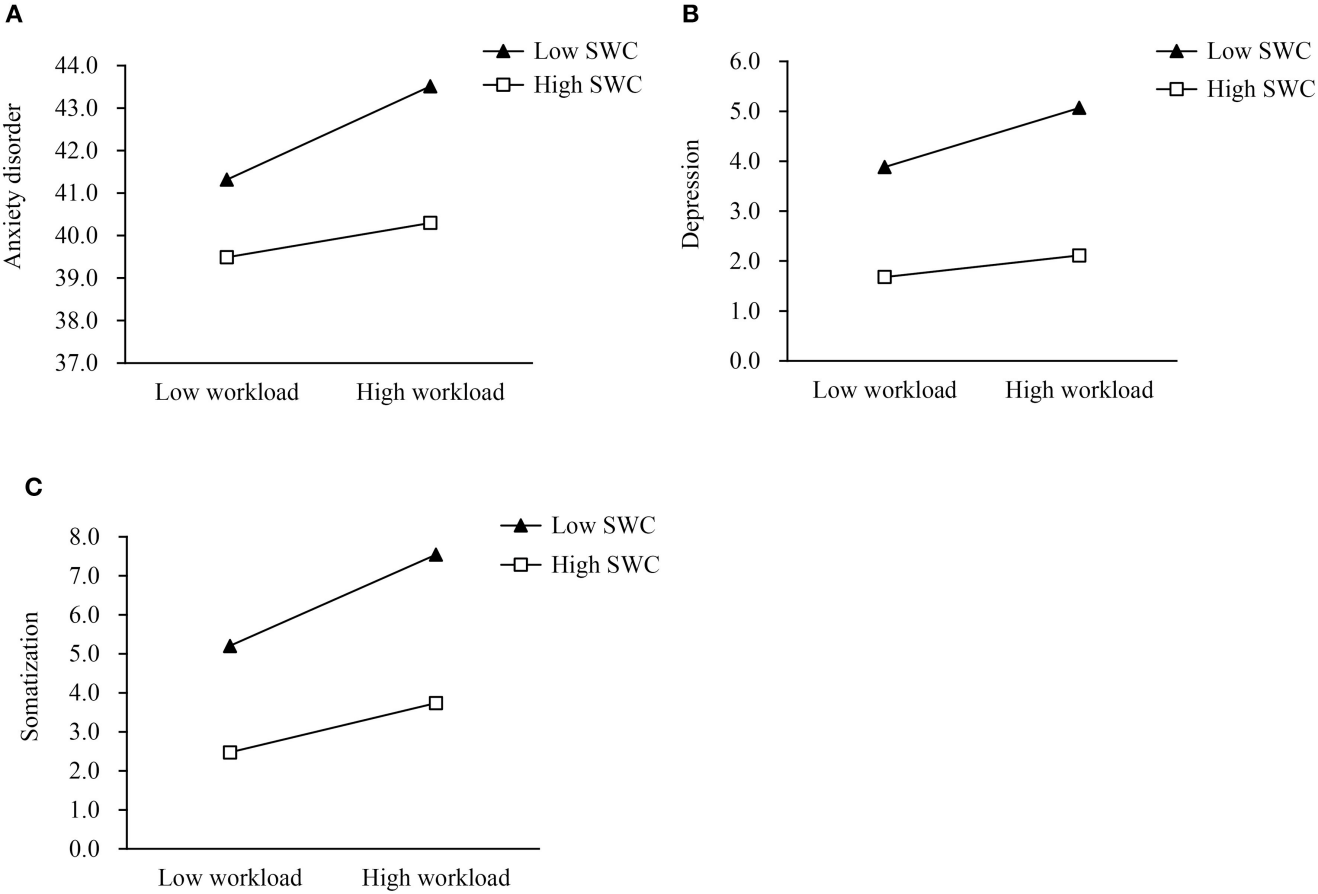
The simple slope of satisfaction with working conditions moderated the associations between workload and anxiety disorder **(A)**, depression **(B)**, and somatization **(C)**. SWC, Satisfaction with working conditions.

Figure 2 shows the simple slope of SWC moderating the association between workload and anxiety disorder, as well as depression and somatization. The results suggested that for healthcare workers with low SWC, the high workload was associated with an anxiety disorder (*b*_simple_ = 1.10, *p* < 0.001) (Supplementary Table 1; Figure 2A). However, for healthcare workers with high SWC, high workload was not significantly associated with an anxiety disorder (*b*_simple_ = 0.40, *p* = 0.134). Similarly, for healthcare workers with low SWC, high workload was associated with depression (*b*_simple_ = 0.59, *p* < 0.001). However, for healthcare workers with high SWC, high workload was not significantly associated with depression (*b*_simple_ = 0.22, *p* = 0.124) (Supplementary Table 1; Figure 2B). Healthcare workers with a high workload were associated with somatization both for low SWC (*b*_simple_ = 1.17, *p* < 0.001) and high SWC (*b*_simple_ = 0.63, *p* < 0.001) (Supplementary Table 1; Figure 2C).

Furthermore, the results in the Johnson-Neyman method showed that the cut-off value for SWC moderating the association between workload and anxiety disorder was 0.79 (Supplementary Figure 1; Supplementary Table 2), which was 0.81 for depression (Supplementary Figure 2; Supplementary Table 3). When the SWC value was below 0.79 and 0.81, the workload was associated with anxiety disorder and depression, respectively, but this effect was no longer significant when the value was above the cut-off values. The workload was always associated with somatization, regardless of the value of SWC (Supplementary Figure 3; Supplementary Table 4).

## 4. Discussion

This study examined the association between workload and the mental (including depression and anxiety disorder) and physical health of healthcare workers during a local outbreak of COVID-19 and explored whether satisfaction with working conditions moderated this association. The results of the study showed that workload was positively related to anxiety disorder, depression, and somatization among healthcare workers collecting test samples, and this effect was moderated by healthcare workers' satisfaction with their working conditions. Understanding the associations among these factors had a heuristic value for public planning and interventions aiming to reduce the negative psychological and physical outcomes of healthcare workers.

In addition, the results also suggested that women reported higher levels of anxiety disorders, depression, and anxiety disorders compared to men but only reached significant levels of somatization.

The findings suggested that women were more likely to report somatization as compared to men, and that the risk of anxiety and depression was comparable in men and women, which could be explained by the fact that the overall physical fitness of women and the body's ability to bear the load was weaker than that of men (25). It was worth noting that nurses were the main staff for nucleic acid sample collection and that the majority of the composition of this group was women. Similarly, healthcare workers who had participated before in the first-line anti-epidemic reported a higher risk of somatization, which indicated that the burden on the body from previous intense workloads had not been fully recovered and that the current workload might exacerbate this negative effect. These findings have highlighted the need for providing mental and physical health services for healthcare workers, especially for those women and participants who participated in the first-line anti-epidemic before, and it was essential to provide more somatic services and adequate rest to promote somatic health.

The results also showed that healthcare workers aged 26–35 years reported a higher risk of higher anxiety disorder. The possible reasons for this were that healthcare workers in this age group had just entered the workforce and were on the rise in their careers, thus health concerns and career confusion might have increased their anxiety levels. Compared with college, healthcare workers with undergraduate and graduate education or above have a lower risk of depressive symptoms, as well as somatic symptoms, but a significant difference was only observed in undergraduate education. Studies indicated a U-shaped association between depression and education level, the perceived subjective wellbeing from a graduate degree was much lower compared to getting a college degree (26, 27). In addition, the results from this study suggested higher self-rated health was associated with a lower risk of anxiety disorder, depression, and somatization, which was consistent with previous results (28). Self-rated health is widely accepted as a means of reporting physical health and psychological health (29, 30). These findings suggested that the mental and somatic services provided by health providers need to take into account differences in characteristics.

The findings of the present study indicated that workload was a significant predictor of anxiety disorder, depression, and somatization of healthcare workers collecting test samples. This was consistent with previous studies in which excessive workload during a new coronary pneumonia outbreak was strongly associated with the mental health of medical staff, such as anxiety disorder, depression, insomnia, and somatization (6, 31, 32). Overloads, including extended working hours and high workloads, left healthcare workers without good rest and prone to occupational fatigue, which affected their mental and physical health (33). The work requirement control model theory suggested that the amount of work tasks was an important factor in the characteristics of work requirements and was one of the important sources of work stress (8, 34). Overwork and psychological disorders among healthcare workers were important social issues and have been an important concern for the researcher (35). The COVID-19 pandemic in 2019 was undoubtedly unprecedented and greatly increased the risk of mental health problems, which might last for a longer time, and has highlighted concerns about the mental and physical health of healthcare workers. More health services should be provided to mitigate this challenge.

This study found that a higher level of satisfaction with working conditions negatively predicted the mental problems of healthcare workers. Not only that, but satisfaction with working conditions also mitigated the negative effects of workload on the poor mental health of healthcare workers. The results of this study confirmed the theoretical hypothesis of the J-R model. On the one hand, more resources positively impacted the level of psychological health and reduced emotional exhaustion and negative idleness in healthcare workers, thus improving psychological health. On the other hand, such resources also alleviated the negative effects of work characteristics on psychological symptoms. As Bakker et al. (14) pointed out, adequate work resources could help employees successfully achieve their work goals, motivate their learning and growth, promote their development, and bring positive effects to them. The findings highlighted the importance of providing frontline healthcare workers with adequate medical resources, efficient and good work processes, and working conditions, which were essential for reducing the psychological burden of healthcare workers and mitigating the negative mental health effects of high work intensity.

There are several limitations to this study. First, this study was a cross-sectional investigation, and this kind of study limited the ability of causal inference. Further longitudinal studies are needed to replicate and reproduce these findings. Second, data were collected based on a web-based questionnaire, which could not guarantee the response rate. Finally, the measurement of workload in this study was limited only in terms of shift duration and the number of nucleic acid tests participated in; therefore, future studies should be conducted using more refined instruments to evaluate these associations.

## 5. Conclusion

Overall, this study highlighted the negative impact of workload on the psychological health of sampled healthcare workers in the post-epidemic era, which could be moderated by satisfaction with working conditions. Healthcare workers still faced beyond-neglect psychological challenges, and proactive health service measures should be considered to alleviate these challenges.

## Data availability statement

The raw data supporting the conclusions of this article will be made available by the authors, without undue reservation.

## Ethics statement

The studies involving human participants were reviewed and approved by Zhengzhou University. The patients/participants provided their written informed consent to participate in this study.

## Author contributions

KW: conceptualization, data analysis, writing—original draft, and visualization. BY: methodology, data curation, writing—review, and editing. LZ: investigation, data curation, writing—review, and editing. CW: conceptualization, resources, formal analysis, supervision, and project administration. All authors read and approved the final manuscript.
