# Supplementary material for: The moderation of satisfaction with working conditions in the association between workload and mental health among healthcare workers collecting test samples in the post-COVID-19 era
Source: Front Public Health. 2023 Jun 8;11:1106299. doi: 10.3389/fpubh.2023.1106299 (PMC10286863; doi:10.3389/fpubh.2023.1106299)
Supplement: Supplementary file 1 [file Data_Sheet_1.ZIP › Supplementary materials.docx]

**Table S1** The effect of workload on anxiety disorder, depression, and somatization under different levels of moderating variables(M±1SD)

| Outcome | *SWC* | Effect | *se* | *t* | *p* | 95% CI | |
| --- | --- | --- | --- | --- | --- | --- | --- |
|  |  |  |  |  |  | Lower | Upper |
| Anxiety disorder | *M*-*SD* | 1.10 | 0.291 | 3.783 | ＜0.001 | 0.53 | 1.67 |
|  | *M* | 0.75 | 0.201 | 3.734 | ＜0.001 | 0.36 | 1.15 |
|  | *M+SD* | 0.40 | 0.269 | 1.501 | 0.134 | -0.12 | 0.93 |
| Depression | *M-SD* | 0.59 | 0.152 | 3.887 | ＜0.001 | 0.29 | 0.89 |
|  | *M* | 0.40 | 0.105 | 3.836 | ＜0.001 | 0.20 | 0.61 |
|  | *M+SD* | 0.22 | 0.141 | 1.541 | 0.124 | -0.06 | 0.49 |
| Somatization | *M-SD* | 1.17 | 0.197 | 5.919 | ＜0.001 | 0.78 | 1.56 |
|  | *M* | 0.90 | 0.137 | 6.580 | ＜0.001 | 0.63 | 1.17 |
|  | *M+SD* | 0.63 | 0.182 | 3.450 | ＜0.001 | 0.27 | 0.99 |

Abbreviations: *SWC*=Satisfaction with working conditions; *M=*Mean; *SD=*standard deviation.


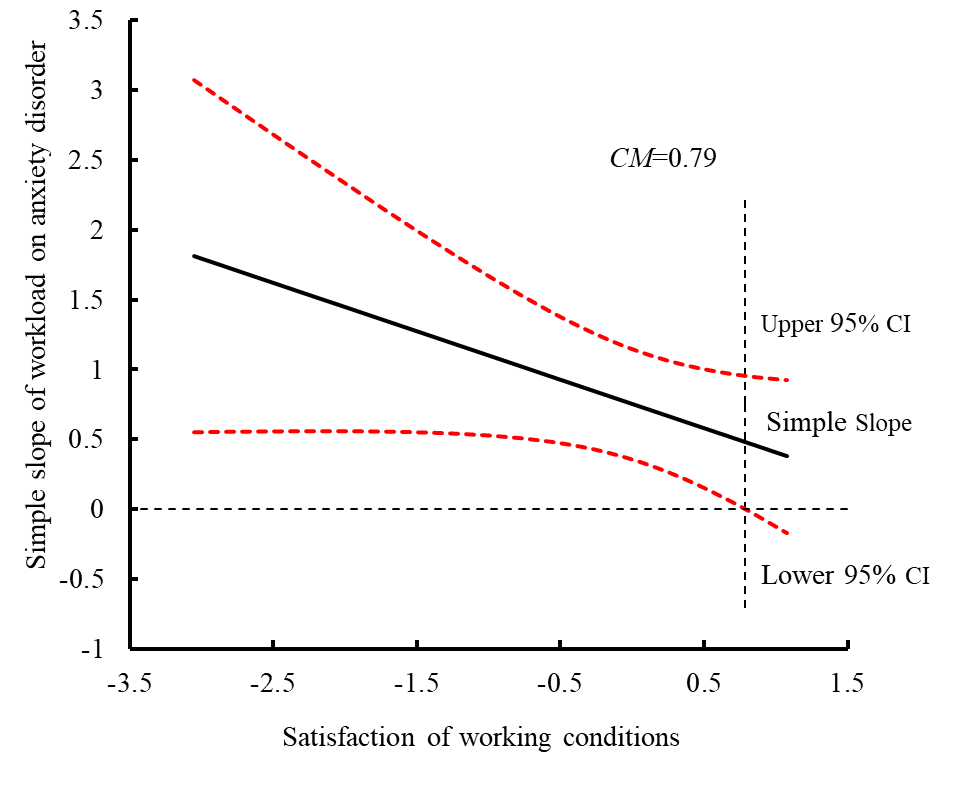


**Fig. S1** The changing trend of moderating effect of satisfaction with working conditions on the relationship between workload and anxiety disorder. Abbreviations: CI= Confidence Interval.

**Table S2** Satisfaction with working conditions moderated the association between workload and anxiety disorder.

| *SWC* | Effect | 95%CI | |
| --- | --- | --- | --- |
|  |  | Lower | Upper |
| -3.05 | 1.81 | 0.55 | 3.07 |
| -2.85 | 1.74 | 0.56 | 2.93 |
| -2.64 | 1.67 | 0.56 | 2.78 |
| -2.44 | 1.60 | 0.56 | 2.64 |
| -2.23 | 1.53 | 0.56 | 2.49 |
| -2.02 | 1.45 | 0.56 | 2.35 |
| -1.82 | 1.38 | 0.56 | 2.21 |
| -1.61 | 1.31 | 0.56 | 2.07 |
| -1.40 | 1.24 | 0.55 | 1.93 |
| -1.20 | 1.17 | 0.54 | 1.79 |
| -0.99 | 1.10 | 0.53 | 1.66 |
| -0.78 | 1.02 | 0.51 | 1.54 |
| -0.58 | 0.95 | 0.49 | 1.42 |
| -0.37 | 0.88 | 0.45 | 1.31 |
| -0.16 | 0.81 | 0.40 | 1.21 |
| 0.04 | 0.74 | 0.34 | 1.13 |
| 0.25 | 0.67 | 0.27 | 1.06 |
| 0.46 | 0.59 | 0.17 | 1.01 |
| 0.66 | 0.52 | 0.07 | 0.98 |
| **0.79** | **0.48** | **0.00** | **0.96** |
| 0.87 | 0.45 | -0.05 | 0.95 |
| 1.08 | 0.38 | -0.17 | 0.93 |

Abbreviations: *SWC=*Satisfaction with working conditions;

CI= Confidence Interval.


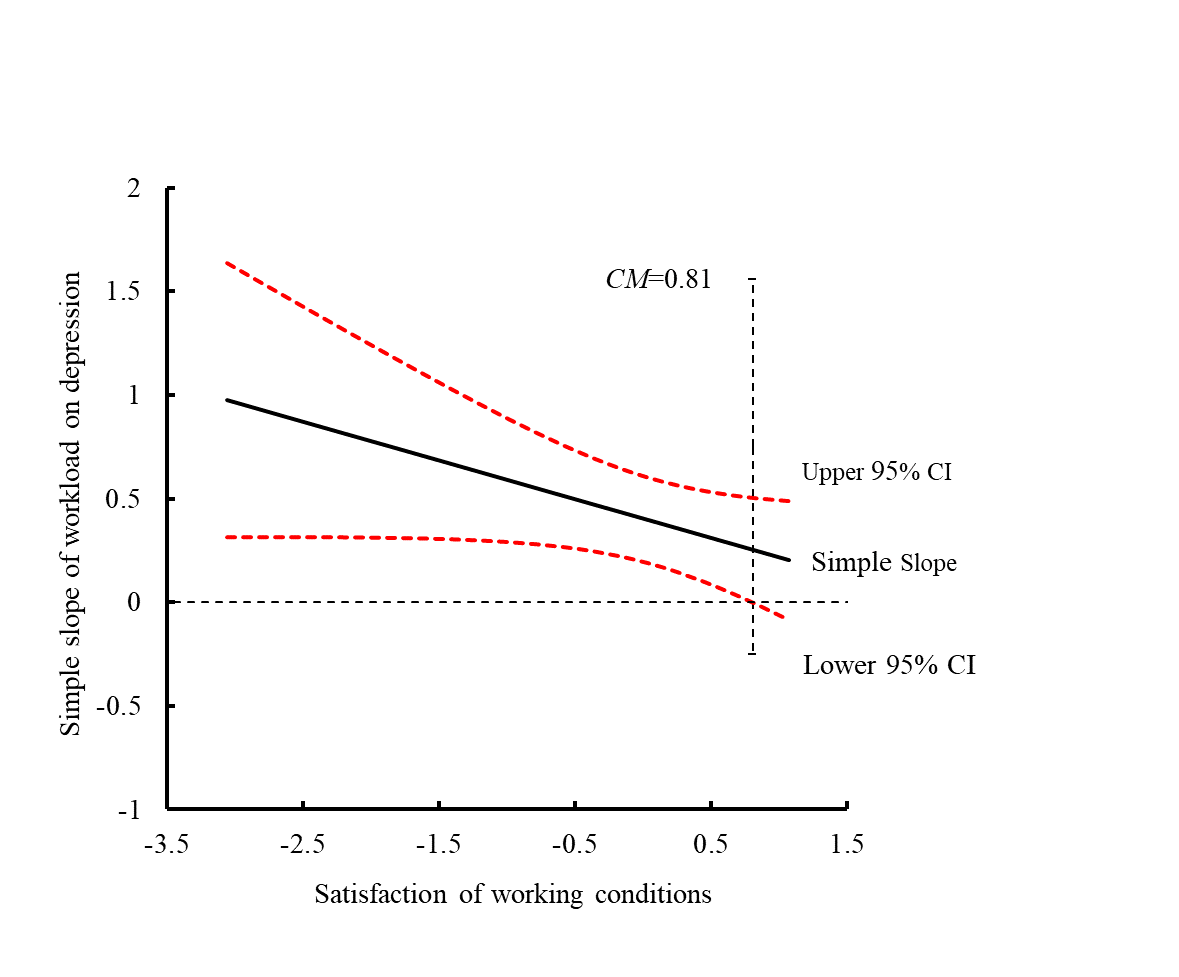


**Fig. S2** The changing trend of moderating effect of satisfaction with working conditions on the relationship between workload and depression. Abbreviations: CI= Confidence Interval.

**Table S3** Satisfaction with working conditions moderated the association between workload and depression

| *SWC* | Effect | 95%CI | |
| --- | --- | --- | --- |
|  |  | Lower | Upper |
| -3.05 | 0.98 | 0.32 | 1.64 |
| -2.85 | 0.94 | 0.32 | 1.56 |
| -2.64 | 0.90 | 0.32 | 1.48 |
| -2.44 | 0.86 | 0.32 | 1.40 |
| -2.23 | 0.82 | 0.32 | 1.33 |
| -2.02 | 0.78 | 0.31 | 1.25 |
| -1.82 | 0.74 | 0.31 | 1.18 |
| -1.61 | 0.71 | 0.31 | 1.10 |
| -1.40 | 0.67 | 0.31 | 1.03 |
| -1.20 | 0.63 | 0.30 | 0.96 |
| -0.99 | 0.59 | 0.29 | 0.89 |
| -0.78 | 0.55 | 0.28 | 0.82 |
| -0.58 | 0.51 | 0.27 | 0.76 |
| -0.37 | 0.47 | 0.25 | 0.70 |
| -0.16 | 0.44 | 0.22 | 0.65 |
| 0.04 | 0.40 | 0.19 | 0.60 |
| 0.25 | 0.36 | 0.15 | 0.57 |
| 0.46 | 0.32 | 0.10 | 0.54 |
| 0.66 | 0.28 | 0.04 | 0.52 |
| **0.81** | **0.25** | **0.00** | **0.51** |
| 0.87 | 0.24 | -0.02 | 0.50 |
| 1.08 | 0.20 | -0.08 | 0.49 |

Abbreviations: *SWC=*Satisfaction with working conditions;

CI= Confidence Interval.


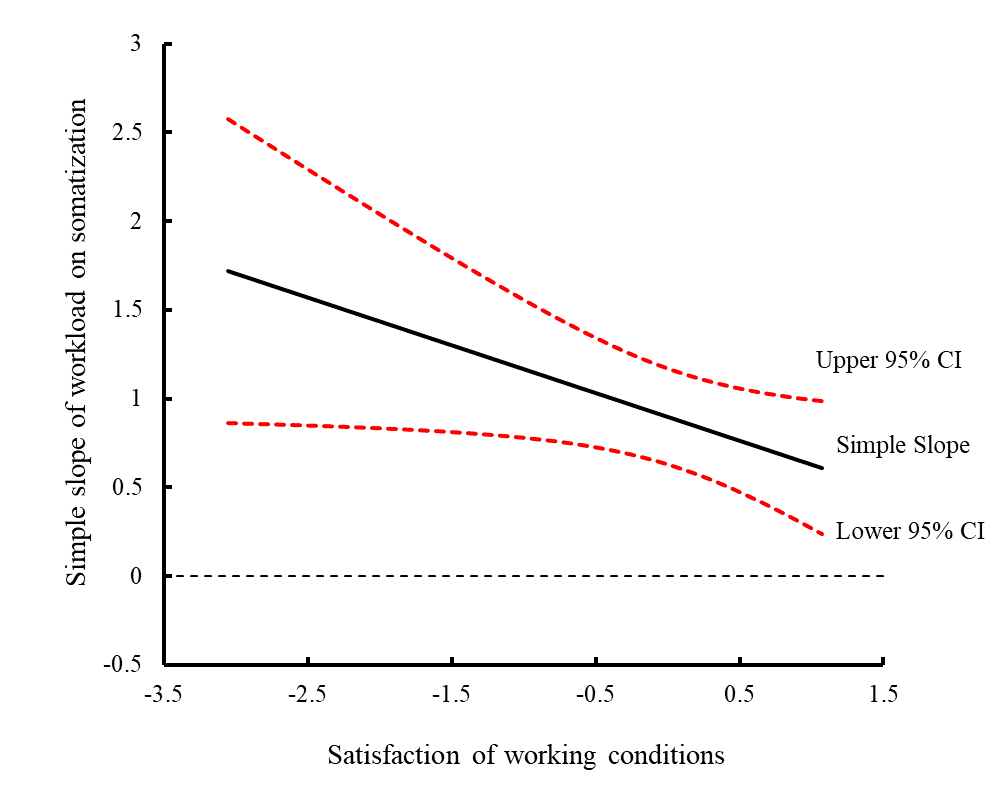


**Fig. S3** The changing trend of moderating effect of satisfaction with working conditions on the relationship between workload and somatization. Abbreviations: CI= Confidence Interval.

**Table S4** Satisfaction with working conditions moderated the association between workload and somatization

| *SWC* | Effect | 95%CI | |
| --- | --- | --- | --- |
|  |  | Lower | Upper |
| -3.05 | 1.72 | 0.86 | 2.58 |
| -2.85 | 1.66 | 0.86 | 2.47 |
| -2.64 | 1.61 | 0.85 | 2.36 |
| -2.44 | 1.55 | 0.85 | 2.26 |
| -2.23 | 1.50 | 0.84 | 2.15 |
| -2.02 | 1.44 | 0.84 | 2.05 |
| -1.82 | 1.39 | 0.83 | 1.95 |
| -1.61 | 1.33 | 0.82 | 1.85 |
| -1.40 | 1.28 | 0.81 | 1.75 |
| -1.20 | 1.22 | 0.80 | 1.65 |
| -0.99 | 1.17 | 0.78 | 1.55 |
| -0.78 | 1.11 | 0.76 | 1.46 |
| -0.58 | 1.06 | 0.74 | 1.37 |
| -0.37 | 1.00 | 0.71 | 1.29 |
| -0.16 | 0.94 | 0.67 | 1.22 |
| 0.04 | 0.89 | 0.62 | 1.16 |
| 0.25 | 0.83 | 0.56 | 1.10 |
| 0.46 | 0.78 | 0.49 | 1.06 |
| 0.66 | 0.72 | 0.42 | 1.03 |
| 0.87 | 0.67 | 0.33 | 1.00 |
| 1.08 | 0.61 | 0.24 | 0.98 |

Abbreviations: *SWC=*Satisfaction with working conditions;

CI= Confidence Interval.
